# Multistate Ferroelectric Diodes with High Electroresistance Based on van der Waals Heterostructures

**DOI:** 10.1021/acs.nanolett.4c03360

**Published:** 2024-10-09

**Authors:** Soumya Sarkar, Zirun Han, Maheera Abdul Ghani, Nives Strkalj, Jung Ho Kim, Yan Wang, Deep Jariwala, Manish Chhowalla

**Affiliations:** † Department of Materials Science and Metallurgy, University of Cambridge, 27 Charles Babbage Road, Cambridge CB3 0FS, United Kingdom; ‡ Department of Electrical and Systems Engineering, University of Pennsylvania, Philadelphia, Pennsylvania 19104, United States; § Center for Advanced Laser Techniques, Institute of Physics, 10000 Zagreb, Croatia

**Keywords:** van der Waals heterostructure, ferroelectric diodes, CuInP_2_S_6_, multibit storage, electroresistance, nonvolatile memory

## Abstract

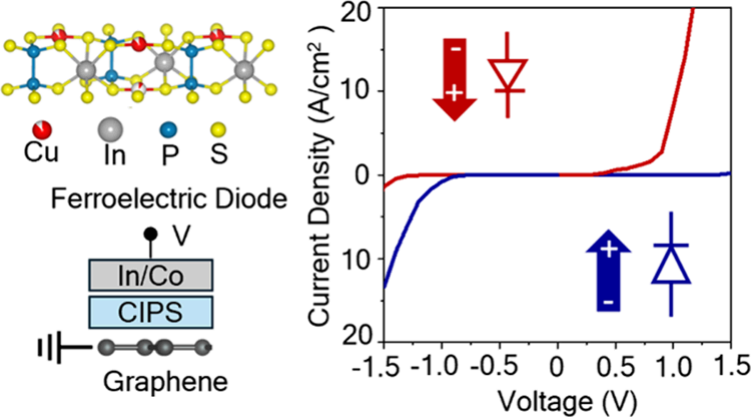

Some van der Waals (vdW) materials exhibit ferroelectricity, making them promising for novel nonvolatile memories (NVMs) such as ferroelectric diodes (FeDs). CuInP_2_S_6_ (CIPS) is a well-known vdW ferroelectric that has been integrated with graphene for memory devices. Here we demonstrate FeDs with self-rectifying, hysteretic current–voltage characteristics based on vertical heterostructures of 10 nm thick CIPS and graphene. By using vdW indium–cobalt top electrodes and graphene bottom electrodes, we achieve a high electroresistance (on- and off-state resistance ratios) of ∼10^6^, an on-state rectification ratio of 2500 for read/write voltages of 2 V/0.5 V, and a maximum output current density of 100 A/cm^2^. These metrics compare favorably with state-of-the-art FeDs. Piezoresponse force microscopy measurements show that stabilization of intermediate net polarization states in CIPS leads to stable multibit data retention at room temperature. The combination of two-terminal design, multibit memory, and low-power operation in CIPS-based FeDs is potentially interesting for compute-in-memory and neuromorphic computing applications.

The rapid advancement of data-driven technologies will require the development of low-power devices that overcome the von Neumann bottleneck for efficient data processing.^1,2^ Nonvolatile memories (NVMs) based on ferroelectric materials represent a promising solution for realizing such integrated devices.^3−6^ Two-terminal devices such as ferroelectric diodes (FeDs) with a metal/ferroelectric/metal vertical heterostructure and resistive readout are promising for high areal storage density and low-power operation.^4,7−10^ Furthermore, due to their rectifying characteristics, FeDs can help alleviate the sneak-path issue without an additional selector circuit or transistor, making their crossbar arrays reliable and energy efficient.^4^ The resistance states in FeDs arise from ferroelectric polarization switching induced modulation of the average barrier height (ABH). This leads to hysteretic leakage current across the ferroelectric layer, which is rectified due to the presence of a Schottky barrier at the metal–ferroelectric interface.

A robust ferroelectric diode with a high electroresistance (defined as the ratio of resistance in the on- and off-states) can provide access to multiple conductance states due to multilevel polarization switching.^11,12^ However, achieving a high on- and off-state (ON/OFF) ratio requires effective modulation of the Schottky barrier height. Excellent FeD performance has been achieved with oxide and nitride ferroelectrics.^7−11,13^ van der Waals (vdW) ferroelectrics, such as CuInP_2_S_6_ (CIPS), which exhibit robust out-of-plane ferroelectricity at room temperature, are also promising for FeDs.^14−17^ vdW materials have a layered structure with weak interlayer bonding, and electronically and atomically sharp interfaces free of dangling bonds—facilitating the formation of heterostructures with other two-dimensional (2D) materials such as graphene.^16,18^ CIPS is a typical vdW ferroelectric in which the ferroelectric polarization is attributed to the displacement of Cu^+^ ions within the sulfur octahedra (see schematic in Figure 1a).^19^ It exhibits intriguing properties such as the coexistence of out-of-plane ferroelectricity and ionic conductivity,^20^ negative piezoelectricity,^21^ and a bulk photovoltaic effect.^22^ Previous reports have demonstrated FEDs with thicker CIPS flakes (>30 nm).^17,23,24^ However, it has been shown that a ferroelectric thickness of ∼10 nm is the optimal regime to combine the effects of barrier height modulation and depletion width modulation to maximize the ON/OFF and rectification ratios.^3,25^

**Figure 1:**
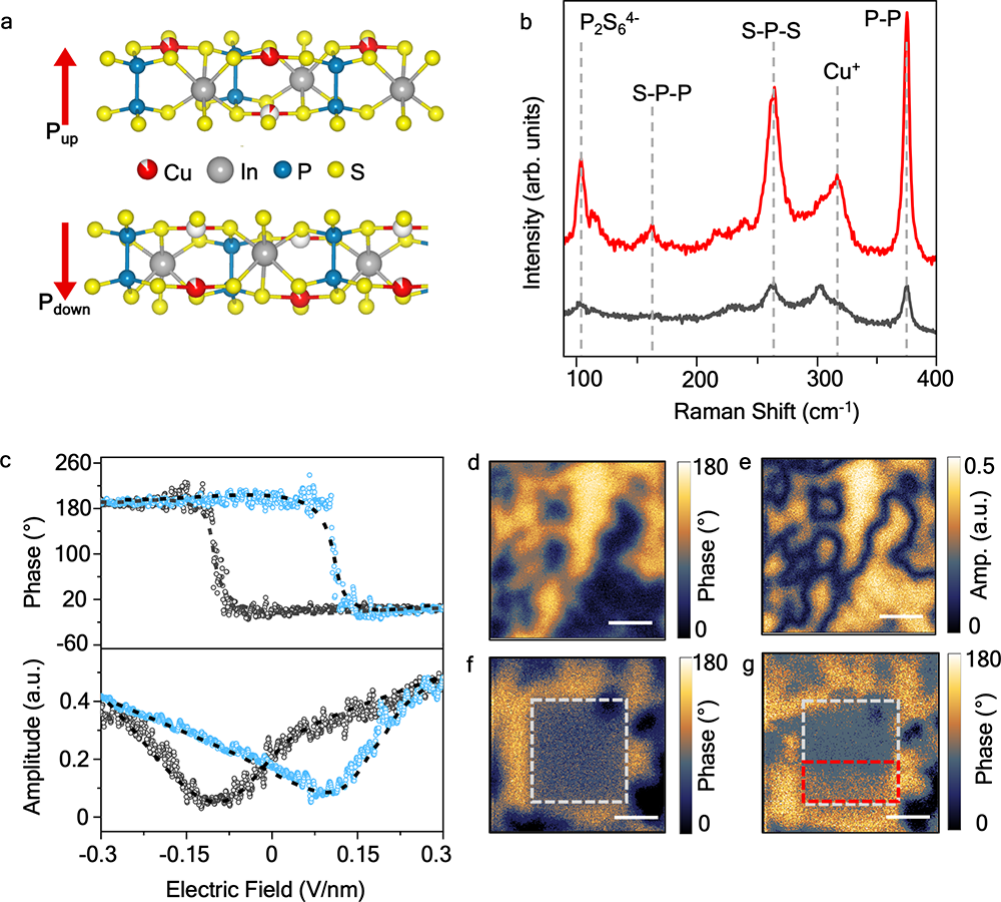
Ferroelectricity in ultrathin CIPS. (a) Schematic of crystal structure of CuInP_2_S_6_ (CIPS) for the two opposite polarization configurations originating from the displacement of the Cu^+^ ion (red). (b) Raman spectra of mechanically exfoliated CIPS flakes on a SiO_2_/Si substrate for flake thicknesses of 45 nm (red) and 8 nm (gray). The characteristic ferroelectric Raman mode (Cu^+^) is detected at 8 nm flake thickness. (c) Out-of-plane piezoresponse force microscopy (PFM) phase and amplitude loops showing characteristic ferroelectric hysteresis measured on a 20 nm thick CIPS/Au test structure. Out-of-plane PFM (d) phase and (e) amplitude maps of the same as-exfoliated CIPS flake show the presence of polar domains. (f) Ferroelectric polarization switching visualized by PFM images after writing a square region with −2.5 V (white box) and (g) +1 V, which causes partial polarization reversal (red box). The scale bar represents 500 nm.

In this study, we fabricated 8–10 nm thick FeDs consisting of metal/CIPS/graphene vertical heterostructures. For the top electrode (TE) we used vdW metal contacts based on an indium–cobalt (InCo) alloy, which minimize defects and Fermi level pinning at the metal–ferroelectric interface to improve carrier injection.^26^ For the bottom electrode (BE), we use graphene, as its Fermi level can be modulated by ferroelectric polarization switching in CIPS.^27^ This asymmetric electrode device architecture allows high ON/OFF (∼10^6^) and rectification ratios (∼2500) to be achieved. The large ON/OFF ratios provide robust noise immunity during operation and enable multiple conductance states (up to 5 demonstrated) that could be useful for multibit computing. Each of these states demonstrates robust retention for up to 100 s. We tested two states which are stable for >1000 switching cycles and can retain data for >10000 s. In addition, FeDs show high readout current densities of 100 A/cm^2^ (maximum) and low read and write voltages of 0.5 and <2.5 V, respectively.

The CIPS flakes were mechanically exfoliated from a bulk crystal and transferred onto prepatterned bottom electrodes on SiO_2_ substrates using a dry transfer technique (as described in the Experimental Section in the Supporting Information). The thickness of the flakes was confirmed via atomic force microscopy (AFM) (see Figure S1 in the Supporting Information). Figure 1b displays the Raman spectra of CIPS on SiO_2_ for flake thicknesses of 45 and 8 nm. The peak at 101 cm^–1^ corresponds to the anionic (P_2_S_6_^4–^) vibrations, while the peaks at 161, 263, and 374 cm^–1^ are attributed to the S–P–P, S–P–S, and P–P modes, respectively.^28^ The peak at 316 cm^–1^, associated with the cationic (In^3+^ and Cu^+^) vibrations, is a signature of the ferroelectric phase.^28,29^ All these modes are present in the 8 nm thick CIPS flake, confirming the ferroelectric crystal structure.

The ferroelectric properties of CIPS were characterized by using piezoresponse force microscopy (PFM). Figure 1c shows the out-of-plane PFM phase and amplitude measurements on a 20 nm thick CIPS/Au/SiO_2_ test device. The clear 180° switching of the PFM phase signal and the well-defined butterfly loops in the PFM amplitude signal indicate robust ferroelectric polarization switching.^17^ Furthermore, the PFM phase and amplitude images of the pristine CIPS sample were mapped (Figure 1d,e), confirming the presence of multiple ferroelectric domains with a lateral size of around 200 nm. The PFM amplitude is constant within the domains and reduced at the domain walls, characteristic of ferroelectric films. The PFM phase reveals domains characterized by two 180°-spaced contrast levels, corresponding to the two opposite polarization directions perpendicular to the film surface. Figure 1f shows the PFM phase images of the same CIPS flake after writing a square pattern (white dashed box) with a negative DC bias of −2.5 V. By applying a positive DC bias of +1 V, we obtain partial reversal of the polarization (phase contrast in the red dashed box in Figure 1g), suggesting stable intermediate net polarization states in CIPS. Such gradual manipulation of ferroelectricity has previously been observed in oxide ferroelectric thin fims.^12^

We investigated the electrical properties of metal/CIPS/metal vertical heterostructures, as shown in Figure 2. The current–voltage characteristics were analyzed for CIPS flakes with thicknesses ranging from 8 to 10 nm, sandwiched between electrodes in a vertical crossbar geometry. The blue plot corresponds to the forward sweep direction from negative to positive bias, and the red plot represents the reverse sweep direction. The BE was electrically grounded for all measurements.

**Figure 2:**
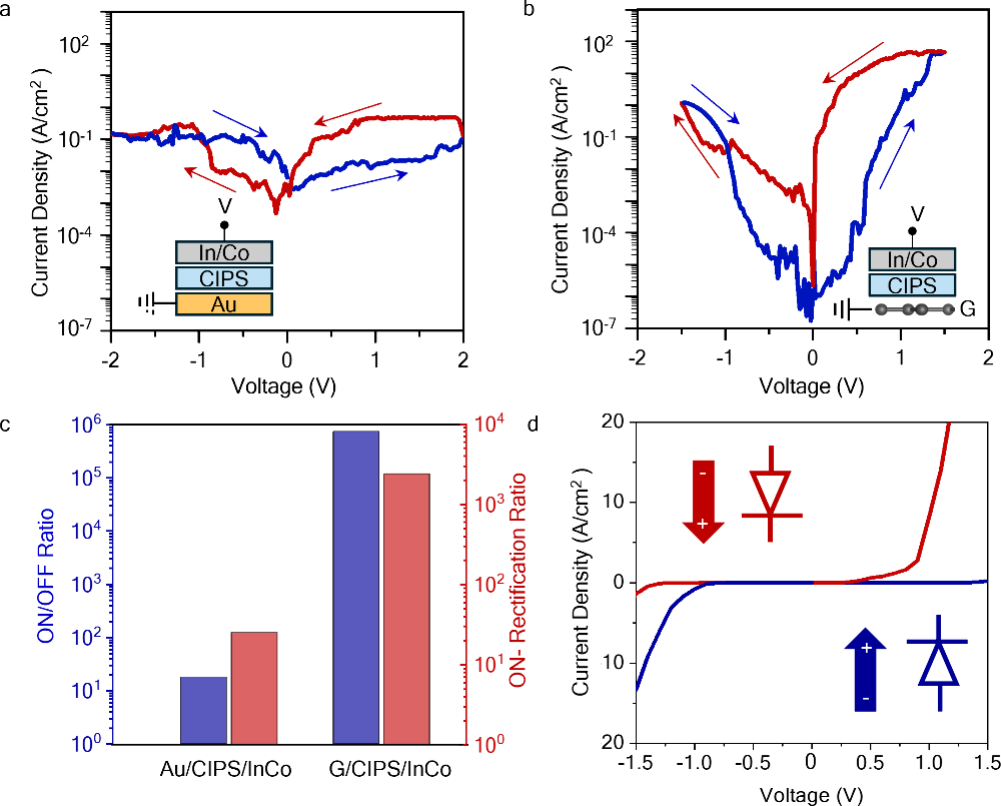
Electrical characteristics of CIPS-based FeDs. Current density vs voltage characteristics measured for two terminal CIPS-based crossbar devices with asymmetric electrodes: (a) Au and In/Co and (b) In/Co and graphene electrodes. The cell size varies from 0.7 to 2 μm^2^. The schematics of the two devices are shown in the inset. The blue and red arrows represent the forward and reverse voltage sweep directions, respectively. (c) ON/OFF ratio (left axis) and on-state rectification ratio (right axis) for the above devices at 0.5 V read voltage. (d) Nonlinear diode-like current–voltage characteristics of a G/CIPS/InCo device with a cell size of 1 μm^2^.

In the first configuration, the BE is a lithographically patterned 20 nm thick Au strip and the TE is a metallic vdW contact based on an In/Co alloy. The In/Co alloy contact was capped with 5 nm thick Au to prevent oxidation (details of the device fabrication process are discussed in the Experimental Section of the Supporting Information). For the Au/CIPS/InCo vertical heterostructure (Figure 2a, with a schematic shown as an inset), hysteretic and nonlinear current–voltage characteristics were observed (the linear current–voltage characteristics are presented in Figure S2 of the Supporting Information). The observed nonlinearity in resistive switching, with an ON state rectification ratio of ∼20 at 0.5 V read voltage, can be attributed to the polarization-related changes in ABH due to asymmetric metal contacts (Au vs In/Co). Resistive switching is not observed above the Curie temperature of CIPS (see Figure S3 of the Supporting Information).

To further investigate the influence of asymmetric Schottky barriers at the interface, we used graphene strips as the bottom electrode (BE) and In/Co as the top electrode (TE). The devices were fabricated by mechanical transfer of CIPS flakes on 1- to 2-layer-thick graphene on SiO_2_ (described in the Experimental Section of the Supporting Information). The G/CIPS heterostructures were characterized by Raman spectroscopy and Raman mapping to identify any heterogeneities in the transfer process (Figure S4 of the Supporting Information). For the G/CIPS/InCo vertical heterostructure devices (Figure 2b, with schematic and OM image in inset), we observed a similar counterclockwise hysteretic current–voltage characteristic, with a giant enhancement in ON/OFF and rectification ratios (the linear current–voltage characteristics are presented in Figure S2 of the Supporting Information). We have presented the ON/OFF ratios and the ON state rectification ratios of the two devices at a low read voltage of 0.5 V in Figure 2c. The variation of ON/OFF ratios as a function of applied voltage is shown in Figure S5 of the Supporting Information. The device with graphene BE has an ON/OFF ratio of approximately 10^6^ at 0.5 V and a rectification ratio of around 2500 (defined as the ratio of the current in the ON state at +0.5 V and −0.5 V). These values are several orders of magnitude higher than those of devices with metallic BEs, and at least an order of magnitude greater than those reported for FeDs based on traditional perovskite oxides or HfO_2_.^25,30^ Furthermore, the current density in the OFF state is reduced in our G/CIPS/InCo FeD devices in comparison with Au/CIPS/InCo FeD devices, suggesting transport is limited at the G/CIPS interface. A high readout current density of 100 A/cm^2^ is measured in the ON state, which is advantageous for high-frequency readout when displacement currents, which scale with frequency, typically become dominant.

The current–voltage curve (in linear scale) of the FeD shown in Figure 2d represents the characteristics of a diode. When a negative voltage of −2 V is applied to the top Co/In electrode, the net polarization points upward. After sweeping to positive voltages by applying a forward bias of +2.5 V, the current changes from low to high. The dipoles are rearranged so that the net polarization points downward and the diode polarity shifts from an OFF-forward diode to an ON-forward diode (red line). The change in polarization from upward to downward direction represents the FeD switching from the OFF state to the ON state. When the voltage sweep direction is reversed toward negative voltages, the diode polarity reverses from an ON-reverse diode to an OFF-reverse diode (blue line), as shown in the inset.

Figure 3a shows the schematic of the 10 nm thick CIPS-based FeDs, where Schottky contacts are formed at the G/CIPS and CIPS/InCo interface (an optical image of the device is shown in Figure 3b). The observed nonlinearity in our current–voltage characteristics is due to the modulation of the ABH and/or depletion layer width in forward bias and reverse bias regimes. The modulation of ABH is due to the opposite polarization charges at the ferroelectric–electrode interface for the two polarization states which can lead to different conduction mechanisms through the device.^31^ As shown in Figure 3c, the high ON/OFF ratio is related to the graphene electrode. The low quantum capacitance of graphene near the Dirac point allows for effective modulation of its Fermi level as a result of ferroelectric polarization switching in CIPS.^27^ In the ON state, the polarization of CIPS points toward the graphene BE and modifies graphene from an intrinsic to an n-type semiconductor, raising the Fermi level above the Dirac point (left side of Figure 3c). This increase in the Fermi level reduces ABH, making it easier for electrons to propagate through CIPS, resulting in higher current. The thickness ∼10 nm of the CIPS layer means that direct tunneling is limited, and conduction across the ferroelectric can occur through defect-assisted tunneling or thermionic injection of carriers above the barrier.^32^ Based on the high output current in the ON state, the dominant conduction can be accounted for by Poole–Frenkel (PF) tunneling—as confirmed by the fit using PF model (see red curve in Figure 3d, and description of the fitting model in the Experimental Section of the Supporting Information).^11,13^ The PF conduction mode is likely enabled by the presence of point defects in the CIPS crystal. We additionally observe that the ON current saturates at larger biases, and this behavior can be attributed to high-field saturation in the PF mechanism.^33^

**Figure 3:**
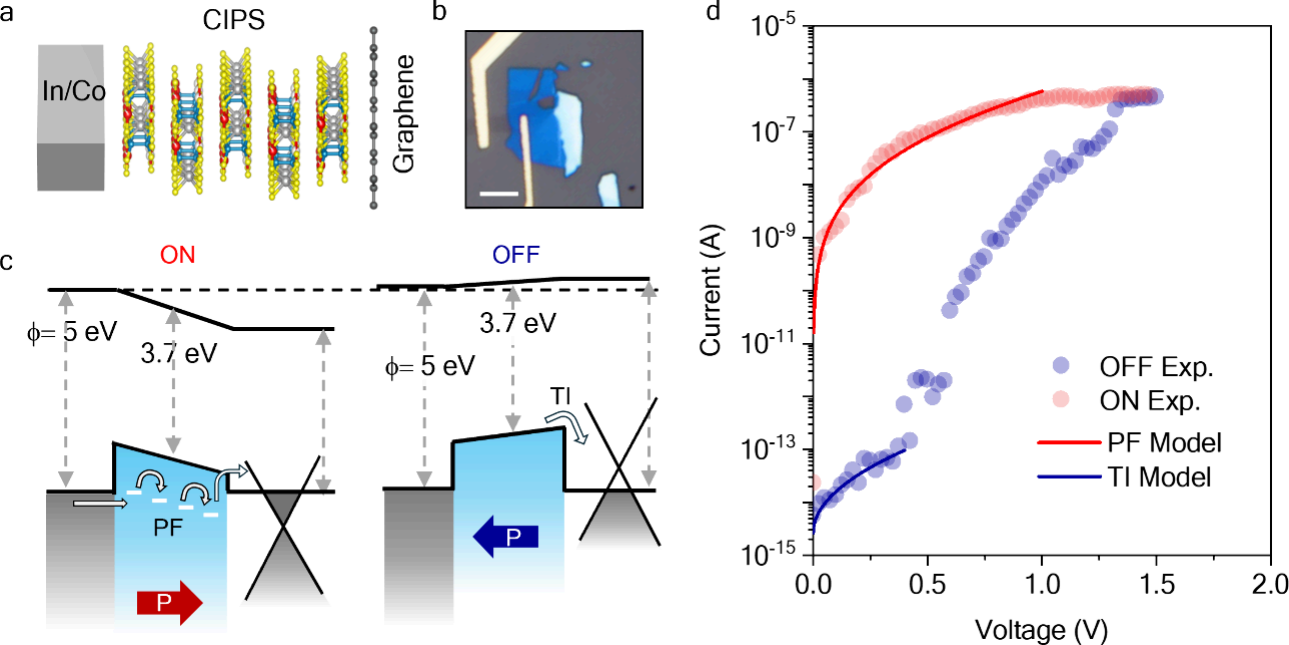
Transport mechanism in CIPS based FeDs. (a) Schematic structure and (b) optical image of the G/CIPS/InCo FeD. The scale bar is 5 μm. (c) Energy band diagrams at zero applied bias and active transport mechanisms of the FeD for ON (left) and OFF state (right). The diagram shows the ferroelectric polarization switching induced ABH modulation and dominant transport mechanisms. (d) Current–voltage characteristics of the FeD fitted with the Poole–Frenkel (PF) and thermionic emission (TI) models.

In the OFF state, the polarization in CIPS points in the opposite direction, shifting the Fermi level of graphene below the Dirac point to the p-type (right side of Figure 3c). This increases the ABH and leads to a low OFF state current (∼10^–14^ A). From Figure 3d, we attribute the interface-limited thermionic emission (TI) over the G/CIPS barrier to the current limiting factor in the OFF-state current. The transport mechanism in the negative voltage regime of the FeD and the Au/CIPS/InCo FeD is described in Section 6 of the Supporting Information.

The current–voltage characteristics of the FeD (shown in Figure 4a, and additional devices as shown in Figure S7 of the Supporting Information) indicate that the transition between the OFF state and the ON state has a low switching slope (∼0.25 V/dec), suggesting a gradual polarization reversal in the CIPS. As shown in the PFM images in Figure 1d,e, the ferroelectric domain size in pristine CIPS is around 200 nm, which suggests the presence of multiple polar domains in a memory cell of area 1–2 μm^2^. Therefore, on applying subcoercive voltages, intermediate net polarization states can be stabilized due to different domain populations as shown by PFM in Figure 1g. Such intermediate polarization states can be reflected as multiple resistance states to store information.^12^

**Figure 4:**
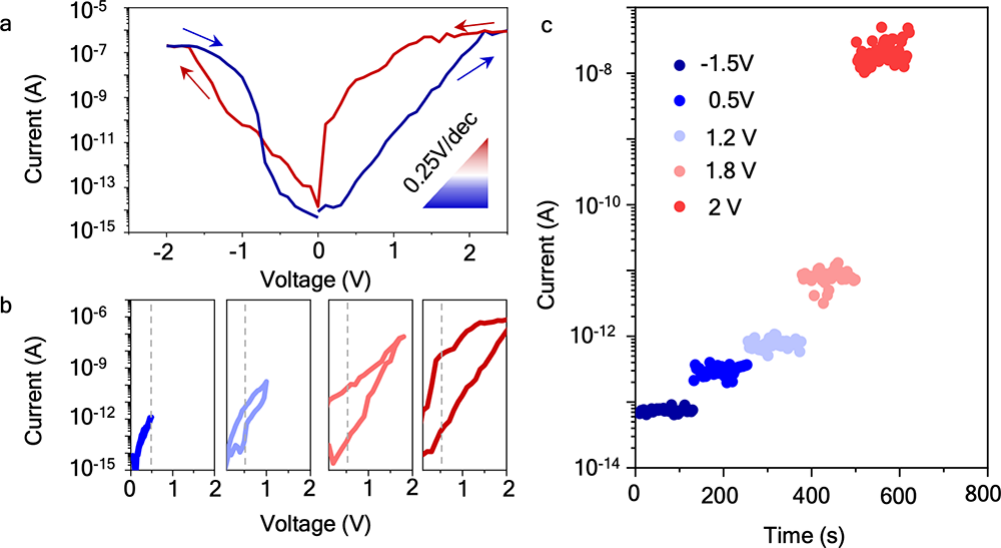
Multiple retention states in CIPS based FeDs. (a) Current–voltage characteristics of the FeD showing a gradual polarization switching from the OFF state (blue) to ON state (red). (b) Current–voltage characteristics measured across a voltage sweep range of 0–0.5, 1, 1.8, and 2.2 V showing the emergence of hysteresis due to partial polarization switching. Prior to these sweeps, the FeD was reset to the OFF state by applying −1.5 V. (c) Multistate retention of the FeD for over 100 s for 5 states measured at 0.5 V read voltage (gray dashed line in (b)).

To stabilize these intermediate resistance states, we gradually increase the voltage scan range from 0–0.5 V, 0–1 V up to 2.5 V. As shown in Figure 4b, the memory window related to ferroelectric hysteresis increases as the scan range increases, allowing us to read multiple intermediate nonvolatile resistance states at a read voltage of 0.5 V (gray dashed line). In Figure 4c, we demonstrate multistate retention for over 100 s for five stable intermediate resistance states, although the state separation would clearly allow for a higher number of distinguishable states. These multiple resistance states are related to the different net polarization states in CIPS.

Figure 5a illustrates the data retention of two of these resistance states. Prior to measuring the retention, the device was first set to the OFF/ON state by applying a write/erase voltage of −2/+2.5 V respectively. Subsequently, the data were read with a bias of 0.4 V. The ON/OFF ratio of the device remained above 10^5^ after more than 10000 s, demonstrating potential for achieving long data retention times in both the ON and OFF states.

**Figure 5:**
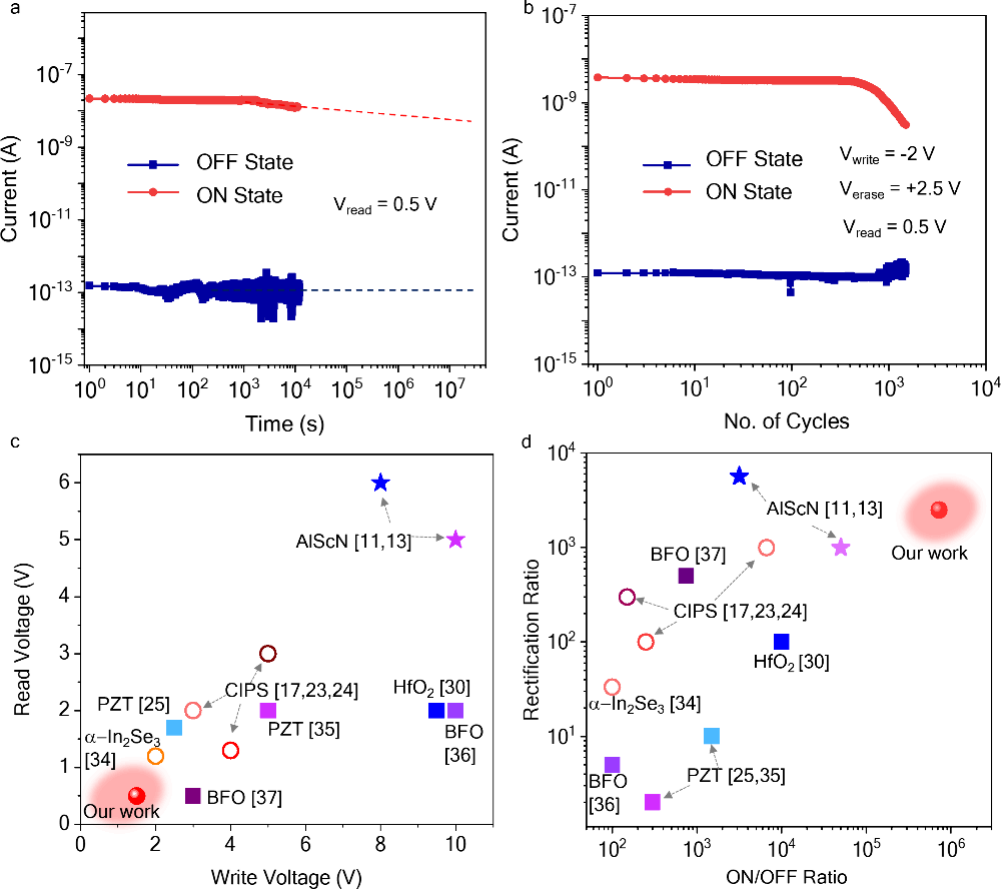
Memory performance of CIPS based FeDs. (a) Two state data retention measurements of 10 nm thick CIPS based FeD for 12000 s. (b) Endurance characteristics of the device. Experimental measurements were obtained for >1500 switching cycles. Comparison of the (c) read voltage vs write voltage and (d) rectification ratio vs ON/OFF ratio of CIPS-based FeDs with previous reported FeDs in literature. The measurements were performed at room temperature.

In Figure 5b, we show the endurance measurement on this FeD over 1500 cycles. Within each cycle, the FeD is set to the ON state by a +2.5 V pulse (10 ms; the pulse shape is described in Figure S8 of the Supporting Information) and read at a bias of 0.5 V, then set to the OFF state using a −2 V pulse (10 ms) and read at a bias of 0.4 V. The ON/OFF ratio remains above 3000 after 1000 write/erase cycles, and the current–voltage characteristics of the device post endurance measurement are shown in Figure S9 of the Supporting Information.

A comparison of the read and write voltages and ON/OFF ratio and rectification ratio of our CIPS-based FeDs with those of previously reported FeDs is presented in Figure 5c,d.^7,8,13,17,20,24,30,34−38^ The sample thickness for these reports and comparison with 3 CIPS-based FeDs is presented in Table S1 of the Supporting Information. The electrical characteristics of our devices compares favorably with those of other reports. The low read and write voltages can be attributed to the use of thinner CIPS samples, and the high ON/OFF and rectification ratios are related to the use of a graphene bottom electrode. Furthermore, the memory performance is improved over previous FeDs based on vdW ferroelectrics due to the use of ultraclean vdW metal contacts that helps minimize defects at the metal and ferroelectric interface. The electrical properties of all our devices as compared to FeDs reported in the literature are summarized in Section S6 of the Supporting Information.

In summary, we have demonstrated FeDs based on vertical heterostructures of layered ferroelectric CIPS with graphene and In/Co vdW metal contacts. The high electroresistance and rectification ratios observed in our devices are attributed to the efficient polarization-modulated transport at the G/CIPS contact interface. Our results indicate that vdW ferroelectric diodes are promising for the design of high-density, selector-free embedded NVMs capable of efficient data processing. We have demonstrated stable data retention over multiple states, which could be beneficial for the development of neuromorphic hardware. Neuromorphic computing architectures rely on physical, i.e. in-hardware, implementation of artificial neural networks (ANNs). As is well-known, ANNs operate on weights assigned to a connection between two physical neurons, and these weights are not binary but rather analog in nature which needs updating as the network “learns” the training data. In this case, having an analog or multibit nonvolatile memory device such as a FeD that can store these weights as programmable resistance values would present a transformative leap in hardware design and implementation. In the future, lateral scaling of devices and large-area, thickness-controlled scalable growth strategies of the CIPS material will be necessary for advancing this promising device concept into a memory technology.

## Supporting Information Available

Experimental details, atomic force microscopy (AFM) images of G/CIPS device, linear current–voltage characteristics of FeDs, Raman spectroscopy of G/CIPS vdW heterostructures, voltage dependence of ON/OFF ratios, conduction mechanism of FeDs, and comparison with previously reported FeDs (PDF)
