# Supplementary material for: Multistate Ferroelectric Diodes with High Electroresistance Based on van der Waals Heterostructures
Source: Nano Lett. 2024 Oct 9;24(42):13232–7. doi: 10.1021/acs.nanolett.4c03360 (PMC11503766; doi:10.1021/acs.nanolett.4c03360)
Supplement: Supplementary file 1 — nl4c03360_si_001.pdf [file nl4c03360_si_001.pdf]

Supporting Information

Multistate ferroelectric diodes with high electroresistance based on van der Waals heterostructures

Soumya Sarkar[1], Zirun Han[2], Maheera Abdul Ghani[1], Nives Strkalj[3], Jung Ho Kim[1], Yan Wang[1], Deep Jariwala[2], Manish Chhowalla[1*]*

[1]Department of Materials Science and Metallurgy, University of Cambridge, 27 Charles Babbage Road, Cambridge CB3 0FS, United Kingdom

[2] Department of Electrical and Systems Engineering, University of Pennsylvania, Philadelphia, Pennsylvania 19104, United States

[3]Center for Advanced Laser Techniques, Institute of Physics, 10000 Zagreb, Croatia

[*]Correspondence should be sent to ss2806@cam.ac.uk and mc209@cam.ac.uk

EXPERIMENTAL SECTION

Sample preparation

CIPS flakes were micromechanically exfoliated using a Nippon sticky tape from a bulk crystal (HQ Graphene) and transferred to PDMS stamps. Thereafter, they were transferred onto pre-patterned bottom electrodes. The graphene bottom electrodes were mechanically exfoliated from a high-quality bulk graphite crystal using M/H Ultron tape. The substrates were lithographically prepatterned 300 nm SiO_2 coated Si. The CIPS and graphene flakes were identified using optical microscopy, atomic force microscopy and Raman spectroscopy.

Device fabrication

The samples were coated with MMA/PMMA resist and electron-beam lithography was used to pattern the electrodes. Before metal electrode deposition, the electron beam evaporation system was pumped to a base pressure of $<10^{-7}$ Torr. Then, 8-10 nm-thick In was deposited with a low rate of 0.1 Å s^{-1} and 40-nm-thick Co was deposited subsequently. 5 nm-thick Au was deposited to prevent oxidation of cobalt. The device was rinsed with isopropanol after immersing in acetone for lift-off.

Raman spectroscopy

Raman spectra and maps were acquired using a Horiba Evolution confocal microraman spectrometer. The samples were excited with a 532 nm laser whose power was kept below 100 μW. A 2400l/mm grating was used to record the spectra and 600l/mm grating was used to collect the Raman maps.

AFM and PFM

AFM imaging was performed using a Bruker Icon system in ambient conditions using a scan rate of 0.6Hz. The AFM images were analyzed using Gwyddion 2.60 software package to determine height profile. Out-of-plane PFM measurements were carried out on Bruker

Multimode AFM system in the piezo response mode. The PFM amplitude and phase loops are an average of measurements collected over 5 spots.

Electrical Characterization

Electrical transport characteristics were measured using a Keithley 4200A SCS semiconductor parameter analyser system in ultrahigh vacuum (< 1×10^{-5} Torr) in a Lakeshore CRX 6.5K probe station. A pre-amplifier was used with the SCS to measure the low currents in the OFF state.

Transport Model Fitting

The transport modeling of the device is based on the Poole-Frenkel emission and thermionic emission models with following two expressions for current density respectively:

$$J_{P-F} = AE \exp\left(-\frac{q\left(\Phi - \sqrt{qE/\pi\epsilon}\right)}{kT}\right)$$

$$J_{TI} = A^{*}T^{2} \exp\left(-\frac{q\left(\Phi - \sqrt{qE/4\pi\epsilon}\right)}{kT}\right)$$

As $\log \frac{J_{P-F}}{E} \propto \sqrt{E}$ and $\log J_{TI} \propto \sqrt{E}$, the equations are first linearized, then linear regression is performed to find the optimal fitting coefficients. The total electric field is calculated as $E = \frac{V}{d} + \Delta E$ where V is the applied voltage, d is the device thickness, and ΔE is a fitting parameter that accounts for a horizontal shift of the current-voltage characteristics caused by the depolarization field arising from the imperfect screening of spontaneous ferroelectric polarization as well as voltage drop across parasitic capacitances. A sweep through a range of values for ΔE is used to find the best fit.

Section 1: Atomic force microscopy (AFM) images of G/CIPS device

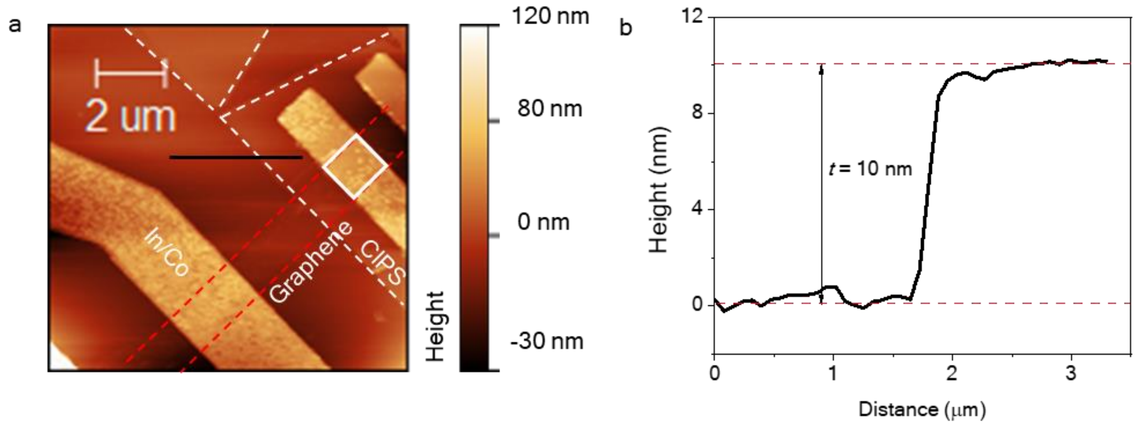

Figure S1 (a) AFM image of G/CIPS/InCo ferroelectric diode (FeD). The CIPS and graphene regions are marked by white and red dashes respectively. The scale bar represents 2 μm. The black line is the region from where the AFM height profile was collected as shown in (b), which indicates the thickness of the CIPS to be 10 nm. The thickness of the graphene was 1-2 layers as confirmed by Raman spectroscopy.

Section 2: Linear current-voltage characteristics of the FeDs

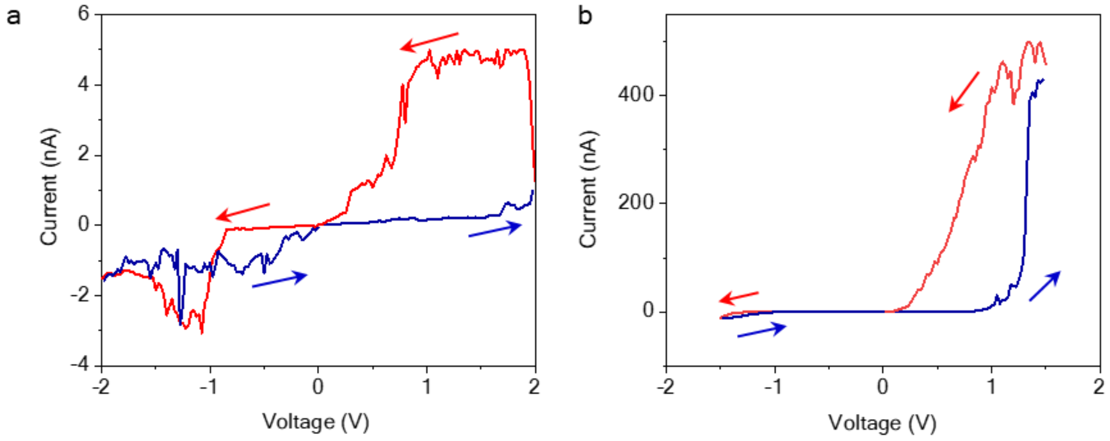

Figure S2 Current-voltage characteristics of (a) Au/CIPS/InCo and (b) G/CIPS/InCo FeDs plotted in a linear current scale. The blue and red arrows represent the forward and reverse voltage sweep directions.

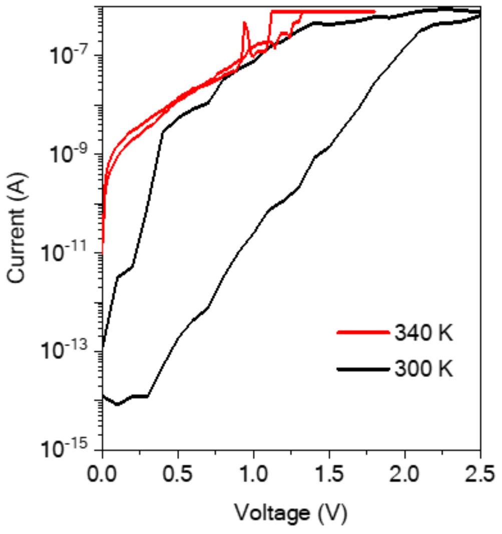

Figure S3 Characteristic current-voltage hysteresis loops of a G/CIPS/InCo FeD measured at room temperature and 340 K.

Section 4: Raman spectroscopy of G/CIPS vdW heterostructures

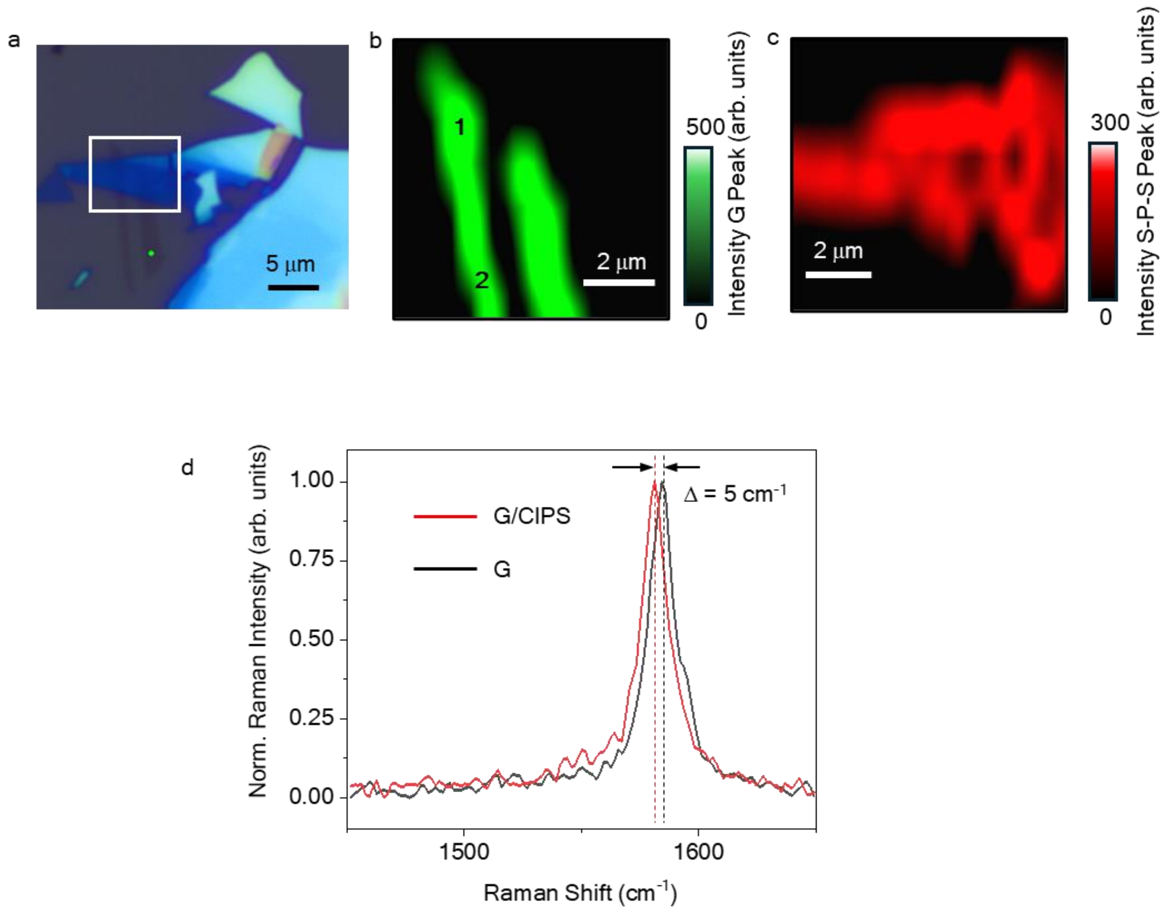

Figure S4 (a) Optical microscope image of a G/CIPS vdW heterostructure. The region in the white box was selected for Raman mapping. (b) Raman intensity map of the (b) G mode in graphene ~1580 cm^{-1} and (c) S-P-S Raman mode in CIPS ~270 cm^{-1}. The uniformity of the intensity of the graphene Raman mode suggests a clean vdW interface post heterostructure fabrication. (d) Raman spectra from the graphene BE collected from the CIPS covered graphene region (spot 1, shown in red spectrum) and bare graphene region (spot 2, shown in black spectrum). The G peak in graphene softens by ~5 cm^{-1} in the CIPS covered region as compared to the bare region.

Section 5 Voltage dependence of ON/OFF Ratios

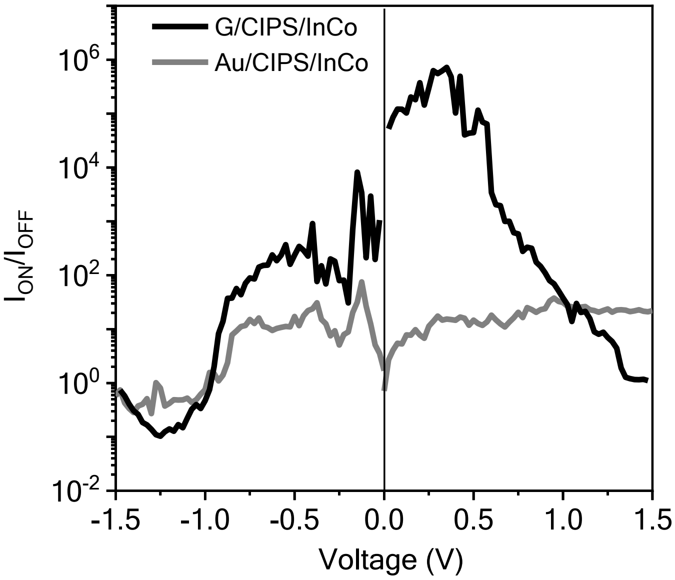

Figure S5 I_{ON}/I_{OFF} of the CIPS based FeDs for two different pairs of electrodes as a function of applied bias. The G/CIPS/InCo FeD exhibits an ON/OFF ratio ~10^6 at bias < 0.5V.

Section 6 Conduction mechanism of the FeD

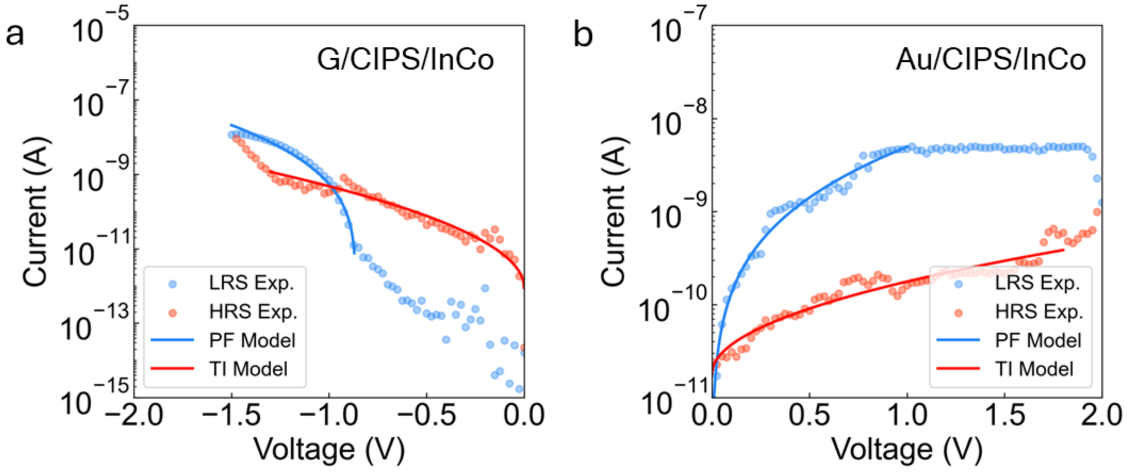

Figure S6 Current-voltage characteristics of the (a) G/CIPS/InCo FeD in the reverse bias regime and (b) Au/CIPS/InCo FeD fitted with the Poole-Frenkel (P-F) and thermionic emission (TI) models.

For negative applied voltages, PF transport is only evident at higher biases, and this can be attributed to the depletion of the graphene due to the P_{up} state of the ferroelectric. A large reverse bias is required to raise the Fermi level above the Dirac point to enable n-type transport described by the PF model.

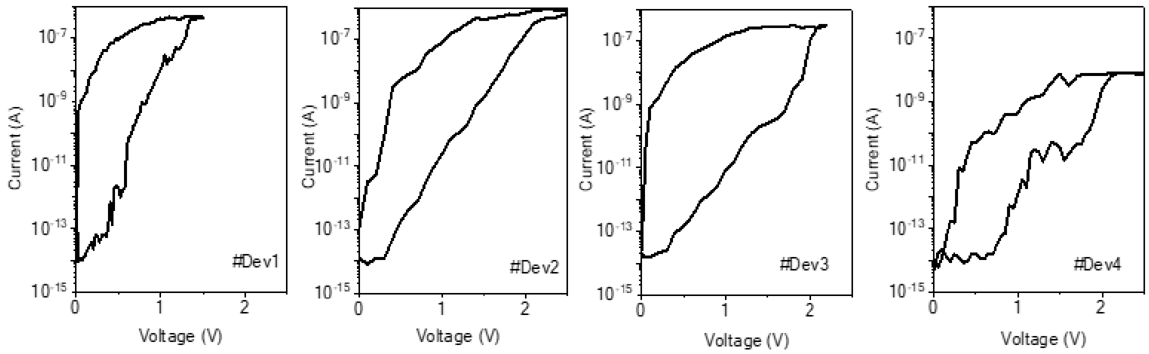

Figure S7 Current-voltage characteristics of 4 InCo/CIPS/G FeDs shows gradual polarization reversal related switching from the OFF state to the ON state. The average power consumption extracted from the current-voltage characteristics of the four devices is 50 femtowatts (in OFF state measured at read voltage of 0.5 V) and 3.7 nW (in ON state, measured at read voltage of 0.5V).

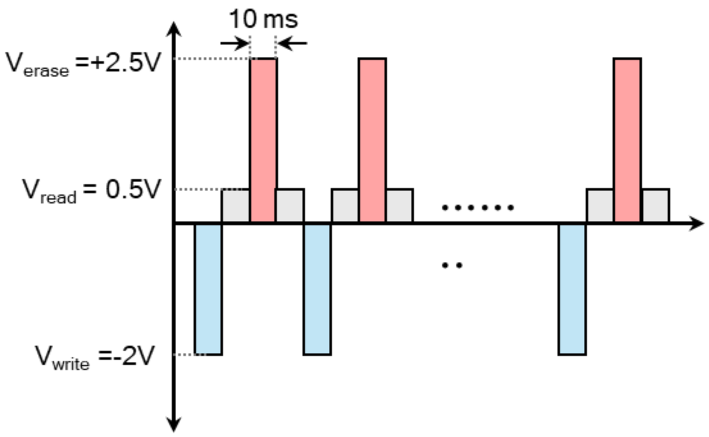

Figure S8 Schematic of the pulse test used for endurance measurements for the InCo/CIPS/graphene FeD.

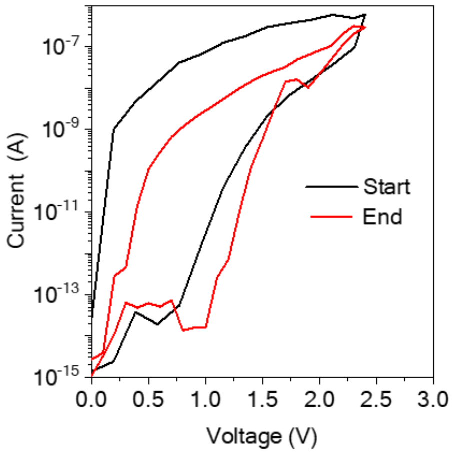

Figure S9 Characteristic current-voltage hysteresis loops of an InCo/CIPS/graphene FeD at the start (black curve) and end (red curve) of the endurance cycles.

Section 7 Comparison with previously reported FeDs

Table S1: A comparison of device characteristics for oxide, nitride, and 2D based FeDs with G/CIPS/InCo based FeD

Sample	Thickness (nm)	ON-OFF Ratio	Rectification Ratio	Read Voltage (V)	Write Voltage (V)
PZT[1]	30	300	2	2	5
PZT[2]	9	1500	10	1.7	2.5
BFO[3]	120	100	5	-2	10
BFO[4]	40	753	200	0.5	3
HfO_2[5]	10	10000	100	2	9.5
AlScN[6]	20	50000	1000	5	10
AlScN[7]	10	3170	5696	6	8
$\alpha-In_2Se_3$[8]	>5nm	100	33	1.2	2
CIPS[9]	120	100	300	3	5
CIPS[10]	167	6637	1000	2	3
CIPS[11]	30	250	100	1.3	4
Our work	10	720864	2500	0.5	1.5
Our work	9	143000	1000	0.5	2.0
Our work	10	90000	1200	0.5	2.5

REFERENCES

(1) Maksymovych, P.; Jesse, S.; Yu, P.; Ramesh, R.; Baddorf, A. P.; Kalinin, S. V. Polarization control of electron tunneling into ferroelectric surfaces. *Science* **2009**, *324* (5933), 1421-1425.

(2) Pantel, D.; Goetze, S.; Hesse, D.; Alexe, M. Room-temperature ferroelectric resistive switching in ultrathin Pb (Zr0. 2Ti0. 8) O3 films. *ACS nano* **2011**, *5* (7), 6032-6038.

(3) Jiang, A. Q.; Wang, C.; Jin, K. J.; Liu, X. B.; Scott, J. F.; Hwang, C. S.; Tang, T. A.; Lu, H. B.; Yang, G. Z. A resistive memory in semiconducting BiFeO3 thin-film capacitors. *Advanced Materials* **2011**, *23* (10), 1277-1281.

(4) Hong, S.; Choi, T.; Jeon, J. H.; Kim, Y.; Lee, H.; Joo, H. Y.; Hwang, I.; Kim, J. S.; Kang, S. O.; Kalinin, S. V. Large Resistive Switching in Ferroelectric BiFeO 3 Nano-Island Based Switchable Diodes. *Advanced Materials* **2013**, *16* (25), 2339-2343.

(5) Luo, Q.; Cheng, Y.; Yang, J.; Cao, R.; Ma, H.; Yang, Y.; Huang, R.; Wei, W.; Zheng, Y.; Gong, T. A highly CMOS compatible hafnia-based ferroelectric diode. *Nature communications* **2020**, *11* (1), 1391.

(6) Liu, X.; Zheng, J.; Wang, D.; Musavigharavi, P.; Stach, E. A.; Olsson, R.; Jariwala, D. Aluminum scandium nitride-based metal–ferroelectric–metal diode memory devices with high on/off ratios. *Applied Physics Letters* **2021**, *118* (20).

(7) Kim, K.-H.; Han, Z.; Zhang, Y.; Musavigharavi, P.; Zheng, J.; Pradhan, D. K.; Stach, E. A.; Olsson III, R. H.; Jariwala, D. Multistate, Ultrathin, Back-End-of-Line-Compatible AlScN Ferroelectric Diodes. *ACS nano* **2024**.

(8) Wan, S.; Li, Y.; Li, W.; Mao, X.; Zhu, W.; Zeng, H. Room-temperature ferroelectricity and a switchable diode effect in two-dimensional α-In 2 Se 3 thin layers. *Nanoscale* **2018**, *10* (31), 14885-14892.

(9) Jiang, X.; Wang, X.; Wang, X.; Zhang, X.; Niu, R.; Deng, J.; Xu, S.; Lun, Y.; Liu, Y.; Xia, T. Manipulation of current rectification in van der Waals ferroionic CuInP2S6. *Nature communications* **2022**, *13* (1), 574.

(10) Li, B.; Li, S.; Wang, H.; Chen, L.; Liu, L.; Feng, X.; Li, Y.; Chen, J.; Gong, X.; Ang, K. W. An electronic synapse based on 2D ferroelectric CuInP2S6. *Advanced Electronic Materials* **2020**, *6* (12), 2000760.

(11) Liu, F.; You, L.; Seyler, K. L.; Li, X.; Yu, P.; Lin, J.; Wang, X.; Zhou, J.; Wang, H.; He, H. Room-temperature ferroelectricity in CuInP2S6 ultrathin flakes. *Nature communications* **2016**, *7* (1), 1-6.
